# A Door-to-Door Prevalence Survey of Dental Fluorosis in Tindigani, a Village in the Hai District of Northern Tanzania

**DOI:** 10.24248/eahrj.v7i2.742

**Published:** 2023-11-30

**Authors:** Claire Stevens, Anna Foat, John Massawe, Ally Mhina, Irene Haule, Daniel Benedict, William K. Gray, Blandina T. Mmbaga, Paul Sallis, Matthew L. Davies

**Affiliations:** a Population Health Sciences Institute, Baddiley-Clark Building, Newcastle University, Newcastle upon Tyne NE2 4AX, United Kingdom; b District Medical Office, Hai District Hospital, Boma Ng'ombe, Hai, United Republic of Tanzania; c Hai District Water Authority, Boma Ng'ombe, Hai, United Republic of Tanzania; d Northumbria Healthcare NHS Foundation Trust, North Tyneside General Hospital, Rake Lane, North Shields NE29 8NH, United Kingdom; e Kilimanjaro Clinical Research Institute, Moshi, United Republic of Tanzania; f Kilimanjaro Christian Medical University College, Moshi, United Republic of Tanzania; g School of Engineering, Newcastle University, Newcastle-Upon-Tyne NE1 7RU, United Kingdom

## Abstract

Fluoride in excess of the World Health Organisation limit of 1.5mg/L in drinking water can cause dental fluorosis (DF) in developing teeth. DF is a significant problem in the Hai District of Northern Tanzania, where there is limited access to safe piped water and groundwater is high in fluoride. A door-to-door prevalence survey of residents of Tindigani village was undertaken to assess current prevalence and severity of DF in the Hai District, and the effectiveness of previous interventions to promote low-fluoride drinking water, following a prevalence survey in 2009. DF was graded by trained assessors, utilising dental photography, and drinking water sources were sampled for chemical analysis. DF was endemic in the 563 people assessed, with a prevalence of 79.4% (CI=76.1–82.7%). Prevalence and severity were found to be higher in permanent teeth than deciduous teeth. Fluoride concentrations in non-piped water sources ranged from 2.5–38.6mg/L. Despite more households reporting the use of low-fluoride, piped water sources, compared to 2009 (82.8% versus 62%), DF remains a significant problem in Tindigani and other such communities where low-fluoride drinking water is not easily and reliably accessible. Policy makers must prioritise reliable access to low-fluoride water, especially for children as their permanent dentition develops.

## INTRODUCTION

Fluorine, existing as part of fluoride-containing minerals, is estimated to be the thirteenth most abundant element in the Earth's crust^1^. Fluoride helps prevent dental caries through its addition to dental products, such as toothpaste, and, in many countries, to drinking water^2,3^. However, excess fluorine can have negative impacts on health including the development of dental fluorosis (DF)^4,5^ and, at higher levels of consumption, skeletal fluorosis (SF)^6,7^.

DF occurs when chronically elevated fluoride intake during tooth formation interferes with the production of ameloblasts (specialised epithelial cells responsible for enamel production), resulting in hypomineralised enamel and increased porosity^8^. Severity of DF is dependent upon dose, duration and timing of exposure with the greatest risk during the secretory and maturation stages of tooth development^9^.

Normal enamel has a creamy, pearlescent quality. In contrast, enamel affected by DF has an opaque, white appearance. Teeth that are mildly affected display faint white horizontal lines. With increasing severity, these striations merge to form mottled patches affecting a greater proportion of the tooth. In severe cases, pitting and enamel loss occur, leading to structural changes in the affected teeth, which are more susceptible to brown staining, sensitivity, impaired function and dental caries^10-13^.

To prevent DF, the World Health Organisation (WHO) recommends a maximum fluoride level in drinking water of 1.5 mg per litre^14^. Globally, several areas are affected by groundwater with high fluoride levels, and endemic fluorosis affects over 25 countries^15^.

In low-income countries, there is often a lack of safe, piped water and, instead, groundwater is used for drinking and cooking. Currently, 75% of Africa's population relies on groundwater as their main source of potable water. This figure is expected to increase, owing to population growth and climate change^16-18^. Groundwater in the East African Rift Valley, which passes through Tanzania, is high in fluoride owing to the underlying volcanic rock composition^19^. Previous research suggests that up to 90% of the Rift Valley population are affected by DF^20,21^. In one study, undertaken in Tanzania, fluoride levels of up to 26mg/l were found in river water and up to 63 mg/l In 2006, a pilot study was conducted examining DF and SF in children attending school in two villages, Mtakuja and Tindigani, located in the southwest of Hai District, Kilimanjaro Region, Tanzania.^24^ The area is affected by low rainfall and communities here, therefore, rely on water high in fluoride from wells or boreholes. Shorter et al. found that the majority of children were affected by DF and more than a quarter had leg deformities consistent with SF.

Following on from the work of Shorter el al., further research, looking at DF and SF, was carried out in Tindigani during 2009.^25^ They carried out a door-to-door survey to establish the prevalence of the conditions in the local population.^25^ Of the 1,207 individuals examined, 911 were found to have some level of DF, a prevalence of 75.5%.

We gather, from the Tindigani village chairman, that following the work of 2 standpipes were installed in central locations within the village to supply safe, lowfluoride drinking water. However, the pipes only operated consistently for a year after installation.^24,25^

### Aims of the Study

The main objective of this research was to measure the prevalence of DF within Tindigani in the south-west of the Hai District, Tanzania.

We also aimed to identify current sources of drinking water, in the area, and measure their fluoride content; assess any changes in drinking-water habits since the study that was conducted in 2009^24,25^, and the impact of such changes on rates of DF; and communicate our findings to the Hai District Water Authority.

## MATERIALS AND METHODS

We conducted a door-to-door, cross-sectional prevalence survey, in which all village residents were eligible to participate.

### Study Design and Location

The study was conducted in the village of Tindigani, which is in the south-west of the Hai District in the Kilimanjaro region of Northern Tanzania. The village is spread over a wide area with no well-defined centre. The majority of the village population belong to the Maasai tribe, with small numbers from other tribes such as the Chagga and Pare. The population is divided into ‘balozis’, family groups, typically, with one male leader. Each ‘balozi’ is further subdivided into ‘bomas’, typically a small group of households led by one male, who may have several wives, and their children. Each ‘balozi’ includes approximately 10 bomas but some may be bigger than this.

As the Maasai typically undertake a form of pastoral farming, sections of the population (especially the older children and middle-aged men) spend significant periods away from home herding their livestock. Consequently, often at least one family member was away when the survey was undertaken. Unfortunately, there was insufficient time available to revisit the affected homes to ensure all family members were included within the study.

### Data Collection

Data collection was undertaken by two masters degree research students over a period of twelve weeks from March to May 2018. They were assisted by two Tanzanian Assistant Medical Officers, who performed translation between Kiswahili and English as necessary, and a village enumerator, who had lived in Tindigani for over 40 years and was able to assist with logistics and translation between Kiswahili and local tribal languages as required. The data collection coincided with the heaviest rains in over fifteen years, which meant that it was not possible to survey the entire village within the available time. No personally identifiable information was linked to the rest of the data. Instead, a separate database was established which included name and study number and only the study number was linked with the main data set. Both databases were stored on password protected computers. For each household surveyed, basic demographic data for the inhabitants, including names, age and sex were recorded. Information was also gathered concerning the current and previous water supplies utilised by the household. Global Positioning System (GPS) coordinates were used to record the location of each household and their corresponding water source. Samples from drinking water sources were collected and analysed for fluoride content using high pressure ion chromatography with standard application checks at Newcastle University. Within each ‘balozi’ one household was chosen at random, to complete a more detailed questionnaire concerning their source of drinking water. The aim of this was to assess the impact of water supplies on the population's day-to-day lives.

Participant's teeth were graded for DF according to the Thylstrup and Fejerskov Index (TFI)^10^. For most participants, grading was carried out using a photograph of their mandibular central incisors, which was taken in natural light and having wiped their teeth dry using gauze. Some individuals consented to participation in the study but not to photography; and these cases were graded in the field. The data collectors had been trained in dental photography and DF grading by a dentist with expertise in assessment and grading of DF.

In addition to the household survey, data were collected from children at the local kindergarten and primary school. This was to ensure that data were captured for the maximum number of children possible. Using this approach, data were gathered for an additional 507 children residing in the village. About 203 children belonged to the households that we had surveyed but who were absent at the time of household data collection, and a further 204 children were from households that were not surveyed.

The main sample comprised data from 1,155 people belonging to 229 households. This consists of data collected at the individual households and from children belonging to those households, gathered whilst they were at school or kindergarten. The data from the 204 children which we were unable to match to a household were analysed separately.

### Ethics

Ethical approval was granted locally from the Kilimanjaro Christian Medical Centre (KCMC) and nationally from the Tanzanian National Institute for Medical Research (NIMR). All individuals involved with the project were offered an information sheet in the relevant language and for those not able to read this was read out for them. The consent form was signed by the head of the household or his or her representative. For individuals not able to write, a thumb print was provided. For those under 16 years consent was obtained from a parent or guardian.

## RESULTS

### Characteristics of Study Population

By comparing our data with contemporaneous census data for Tindigani, it shows that the population included in the household survey comprised 59.4 per cent of the total village population (1,944) and is broadly representative of the entire population in terms of sex and age, showing a pre-demographic transition model. The sample consists of 567(49.1%) males, 588(50.9%) females and 642(55.6%) under the age of 15 year olds. This compares to 989(50.9%) males, 955(49.1%) females and 1,024(52.7%) aged under 15 years, in the village as a whole. The median age of individuals in our sample is 12 years compared to 15 years for the whole village.

Of the 1,155 in the household survey, acceptable quality examination was performed for 563 people. Of those for whom dental examination was not carried out 572 were absent at the time of examination. For 15 people inadequate dental photography precluded DF grading and 5 people had inadequate dentition (central incisors not visible).

**FIGURE 1:**
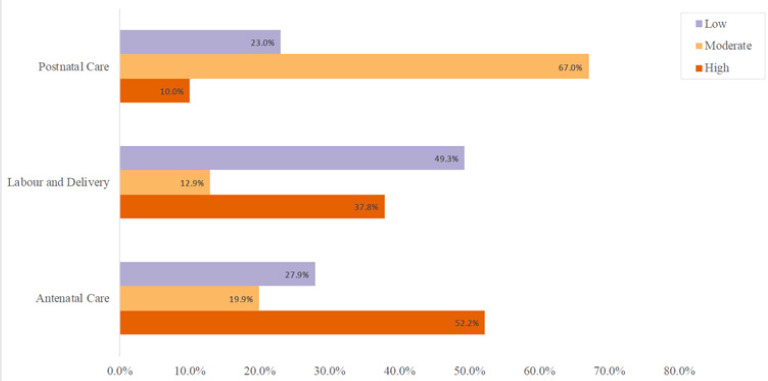
Population Pyramid for Surveyed Households

Note: The grey areas represent the individuals for whom acceptable quality dental examination was carried out. Smaller age ranges were used for the younger children.

Figure 1 shows a population pyramid for the households included in our survey. The proportion of individuals for whom we were able to complete dental examination is represented by the shaded portions. There is considerable variation in these proportions between age and sex. The figure shows that we were generally able to examine fewer men between 15 and 65, as they were more likely to be absent at the time of the survey, typically as a result of attending to livestock.

Of the 563 individuals with acceptable quality examination, 223 were male with a median age of 8, and 340 were female with a median age of 15. Of the 586 individuals in the household survey who were absent or had inadequate examination, 338 were male with a median age of 17, and 248 were female with a median age of 14.

### Sources of Drinking Water

**FIGURE 2:**
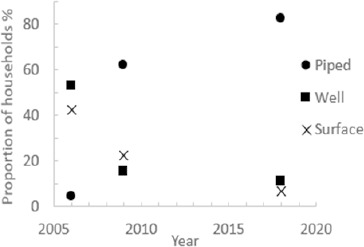
Self-Reported Main Source of Household Drinking Water

Figure 2 shows the primary drinking water sources reported in the household survey compared to previous work carried out in Tindigani^25^. As the figure shows, reliance on well and surface water has decreased significantly since 2006. The proportion of households using well water as their main drinking water source has decreased from 53.1% to 10.9%, and the proportion using surface water from 42.5% to 6.6%. Over the same time period, the proportion of people using piped water as their main supply of drinking water has increased almost twentyfold from 4.4% to 82.5%.

Results from 2006 and 2009 are taken from Jarvis et al.^27^

At the time, the survey undertaken revealed that there was no working supply of piped drinking water to Tindigani itself. However, a number of surrounding villages have standpipes, provided by the Hai District Water Authority, from which water can be collected for a small charge. Of those reporting that they use piped water as their primary water supply, over 95% described using the supply in Sanya Station and the remainder the supply in Rundugai. These villages are approximately six and seven kilometres respectively from Tindigani. Borehole water is currently used for drinking by all the school children throughout the school day in addition to being used for cooking their lunchtime meal.

Collection of water is a time-consuming activity for the residents of Tindigani. Participants in our study reported making, on average, 4.4 (range 1 to 7) trips per week to collect drinking water. The mean journey duration was 3.7 hours, with journeys lasting between 30 minutes and nine hours.

### Fluoride Levels

Samples were taken from 28 water sources utilised by Tindigani residents included in the household survey: three piped, four surface and 21 well water sources. The respective mean fluoride contents for piped, surface and well water sources were 0.82 mg/l (range 0.45 to1.56 mg/l), 3.58 mg/l (range 3.38 to 4.08 mg/l) and 21.11 (range 2.52 to 38.59 mg/l). Fluoride content in the school borehole was measured as 12.58mg/l. This is in excess of safe limits and places the children attending school at increased risk of DF. The village were, therefore, advised not to use this source.

### Dental Fluorosis Overall Prevalence

Despite residents of Tindigani reporting increased usage of low-fluoride, piped drinking water, DF remains endemic in the village. Of the 563 dentate individuals examined as part of the household survey, 79.4% (95% CI, 76.1% to 82.7%) were found to have DF. This global figure masks considerable variation in the prevalence depending on age, with the condition being almost universally present in older children and adults. The prevalence of DF in individuals over eight years of age was 97.8% (95% CI, 96.3% to 99.3%).

### Dental Fluorosis Age-Related Prevalence

**FIGURE 3:**
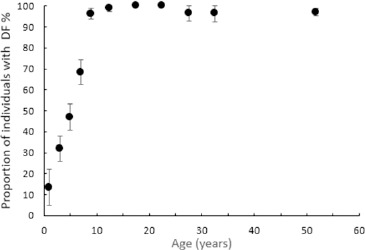
Prevalence of Dental Fluorosis in the Household Survey as a Function of Age in Years. Error Bars Represent the Standard Error of the Mean

Note: The data points have been plotted at the midpoint of each age range except for the one representing the over 35 year olds. As this point encompasses a large age range, it is plotted at the mean age of individuals in that range.

Figure 3 shows the age-related prevalence of DF. Table 1 displays this information in tabular form, along with the mean severity calculated using the TF scores. As can be seen from these data, the prevalence of DF increases significantly from infancy to late childhood by which point almost all individuals are affected.

**TABLE 1:** Age-Related Prevalence and Severity Rates for Household Survey

Age Range (Years)	Dentate Individuals Examined	Cases of DF	Proportion (%) of Dentate Individuals With DF	Mean TF Score in Those With DF
0 – 1	15	2	13.3	5.5 ±1.11
2 – 3	60	19	31.7	2.3 ± 0.3
4 – 5	66	31	47.0	1.8 ± 0.2
6 – 7	60	41	68.3	2.7 ± 0.2
8 – 9	53	51	96.2	3.9 ± 0.2
10 – 14	78	77	98.7	4.7 ± 0.2
15 – 19	36	36	100.0	4.9 ± 0.2
20 – 24	38	38	100.0	5.0 ± 0.3
25 – 29	28	27	96.4	4.9 ± 0.3
30 – 34	27	26	96.3	4.7 ± 0.4
35+	102	99	97.1	4.4 ± 0.2

Abbreviation: DF, Dental Fluorosis; TF, Thylstrup and Fejerskov.

Comparing our data to that of Jarvis et al^25^, we find no significant difference between rates of DF recorded in Tindigani for children under ten years (χ^2^ = 0.892, *P* = 0.345) or in individuals aged 10 to 34 (**χ**^2^ = 0.007, *P* = 0.936). However, using Fisher's exact test, we observe a significant difference in the rate of DF observed in individuals over 35 years (*P* < 0.001). We find a prevalence in this age group of 97.1% (95% CI, 93.8%, 100.0%), whereas Jarvis et al detected DF in 75.5% (95% CI, 73.0%, 77.9%) of subjects in this age range.

**FIGURE 4:**
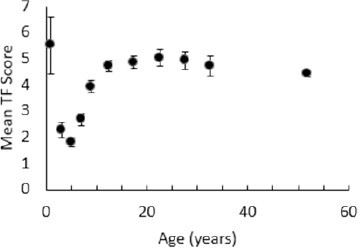
Mean TF Score as a Function of Age in Years for Individuals Affected by Dental Fluorosis

Note: Error bars represent the standard error on the mean. The data points have been plotted at the midpoint of each age range except for that representing over 35 year olds. As this point encompasses a large age range, it is plotted at the mean age of individuals in that range

Figure 4 shows the mean TF score with age for those individuals affected by DF. The graph displays a general trend of increasing severity as childhood progresses, followed by a plateauing at a grade of approximately five upon reaching adolescence. Using Spearman's rank correlation test, we found a significant relationship between age and severity of DF assessed using the TFI for those individuals with DF under 15 years of age (ρ = 0.588, P < 0.001). As noted above, DF severity generally increases with age in this range. Interestingly the general pattern that we have observed is somewhat different to that seen by Jarvis et al. They found a mean TFI score of approximately seven in individuals between 10 and 24 years, with scores falling in older adults to a mean of approximately 4 in adults aged 35 and over.

### Dental Fluorosis Association with Sex

No significant difference was detected in the rates of DF between males and females, either in children under 10 years of age (χ^2^ = 0.216, P = 0.642) or in older individuals (χ^2^ = 0.525, *P* = 0.469). Also, there was no significant evidence for a disparity in the mean TFI between the sexes, for those individuals with DF, either in children under 10 years of age (t = −0.120, P = 0.904) or in those 10 or older (t = 0.242, *P* = 0.809).

### Dental Fluorosis in Deciduous Versus Permanent Dentition

Focusing on children under ten years of age, DF was found to be significantly more common in those with permanent compared to deciduous dentition (χ^2^ = 85.62, *P* < 0.001). In those with deciduous dentition, the prevalence of DF was 35.9% (95% CI, 23.7% to 48.2%) compared to 96.6% (95% CI, 92.6% to100.5%) in permanent dentition.

Of those children in this age group with DF, we observed a significant difference in the mean TFI between those with deciduous and permanent dentition (t = −7.847, *P* < 0.001). The mean TFI was higher in permanent dentition (3.7; 95% CI, to 3.3, 4.0) than deciduous dentition (1.9; 95% CI, 1.6 to 2.2).

### Dental Fluorosis and Household Water Source

There is a significant difference in the rates of DF between those children under ten years of age from households using piped water as their primary water source compared to those using non-piped water (**χ**^2^ = 10.78, *P* < 0.002). Children from homes using piped water had a lower rate of DF (54.8%; 95% CI, 48.0% to 61.5%) compared to those using non-piped sources (65.9%; 95% CI, 51.9% to 79.9%). However, there was no significant difference between the primary sources of water used (piped vs non-piped) by the households of children with DF compared to the households of those without the condition (χ^2^ = 1.84, P = 0.175).

### School and Kindergarten Survey

As part of our research at the local school and kindergarten, we identified 204 children who did not belong to our surveyed households. Of these, we were able to examine 189 individuals, of whom 186 were dentate and 92% (95% CI, 88% to 95.8%) had DF. DF prevalence rates and mean TFI, by age, for these children are shown in Table 2.

**TABLE 2:** Age-Related Dental Fluorosis Prevalence and Severity Rates for School and Kindergarten Survey

Age Range (Years)	Dentate Individuals Examined	Cases of DF	Proportion (%) of Dentate Individuals With DF	Mean TF Score in Those With DF
2 – 3	2	1	50.0	1.0 (–)
4 – 5	44	32	72.7	1.6 ± 0.2
6 – 7	35	33	94.3	2.8 ± 0.3
8 – 9	37	37	100.0	4.4 ± 0.3
10 – 14	67	67	100.0	4.4 ± 0.2
15 –19	1	1	100.0	5.0 (–)

Abbreviation: DF, Dental Fluorosis; TF, Thylstrup and Fejerskov.

The general pattern is similar to that seen for children in the household survey with rates and severity of DF increasing with age, so that the condition is universally present in children over the age of eight years. We note a higher rate of DF in children aged four to seven years in the school and kindergarten survey compared to those in the household survey. There is no significant difference for those children with permanent dentition. However, for those with deciduous dentition, using Fisher's exact test, we detect a significant difference in rates of DF between those children in the school and household surveys in this age group (P < 0.001).

## DISCUSSION

The purpose of this study was to determine the prevalence of DF in the residents of Tindigani village Hai District, whereby the results showed that, 79.4% (95% CI, 76.1% to 82.7%) had DF.

### Water Access and Fluoride Sources

Fluorosis is an endemic issue across much of Tanzania and this is most commonly due to the lack of access to safe piped water with only 57% of the population having close access to a piped water source^26,27^. Due to the scale of the problem in Tanzania, a national fluoride limit of 4mg/l has been set^28^, well above the 1.5mg/l limit recommended by the WHO. This national limit is potentially enough to mitigate the effects of high fluoride concentrations on bone but not on dental development. Although our data reveal that the average fluoride concentration in surface and groundwater in Tindigani is within Tanzanian national guidelines at 3.58mg/l, the only access residents have to water within the WHO recommended limits is through piped water in neighbouring villages, at least 6 km walk away. The UN Committee on Economic, Social and Cultural Rights define the right to water as ‘the right of everyone to sufficient, safe, acceptable and physically accessible and affordable water for personal and domestic uses’ and states that the human right to water ‘is indispensable to leading a life in human dignity and a prerequisite to the realization of other human rights’^29^. The WHO define ‘physically accessible’ as less than 1km from the home and within a collection time of 30 minutes^30^. The right to water is a human right that is not being realised in this, and many other, areas of the world. Not only do surface water, groundwater and well water in Tindigani contain fluoride concentrations significantly above the recommended levels but non-piped water sources are also associated with a higher risk of water-borne diseases such as malaria, cholera and enteric infections^31^. Malnutrition and stunting rates are higher in rural populations without access to piped water^31^.

All samples of well water and surface water had fluoride levels in excess of the WHO–recommended 1.5 mg/l upper limit for drinking water. The mean level detected in well water was over fourteen times this limit and in surface water approximately 2.4 times. On the other hand, the mean fluoride content for piped water was well within the recommended level. Although, one source of piped water had fluoride levels 0.06mg/l above the WHO limit.

Our research shows that there has been a significant change in reported drinking-water practices in Tindigani since 2006. Reliance on piped water as the reported primary source of drinking water has increased markedly with a corresponding drop in use of well and surface water.

Following the work done in 2010 and 2013^24,25^, meetings were held with village residents to improve the awareness of the medical problems associated with drinking water with high levels of fluoride. It could be that this education programme has caused individuals to switch from utilising well and surface water to piped water for drinking. However, we cannot be certain that this is the case without further research into the reasons that people choose to utilise the water sources that they do.

### DF Prevalence and Severity

Unfortunately, despite this shift in practice for over 80% of Tindigani's population, we have not measured a corresponding decrease in the prevalence of DF. As stated above, 79.4% (95% CI: 76.1%, 82.7%) of the dentate individuals examined as part of the household survey were found to have DF.

Jarvis et al. reported the prevalence of DF in Tindigani to be 75.5% (95% CI: 73.0%, 77.9%). This suggests that there may have been an increase in the prevalence of the condition between 2009 and 2018. However, the difference is not statistically significant and our overall prevalence figure from the household survey may not be directly comparable to that of Jarvis et al., since, as noted previously, there was considerable variation in the completeness of our survey between the sexes and various age groups.

The level of completeness in the household survey was lower for working-age males compared to those in younger age groups. Our research shows that DF is almost universally present in the adult population.

Consequently, we would expect our overall prevalence figure to be an underestimate.

An increase in overall prevalence of DF could be explained by the significantly higher levels of DF in adults over 35 than was found by a related study.^24^ A potential reason for the increase in DF amongst this age group is the movement of individuals aged 25 to 34 in 2009 into the older age bracket by 2018. Jarvis et al. found that adults over 35 were less affected by DF than younger adults. The reason for this is unclear.^25^

However, from our discussions with villagers, we know that a number of wells for drinking water were installed by international NGOs in the 1980s and 1990s. It may be that prior to this there was a greater reliance on surface water relative to well water, which we found to have significantly higher average levels of fluoride. This serves to emphasise the importance of organisations installing water supplies conducting adequate prior testing to ensure that they will conform to WHO water-quality guidelines.

We detected no significant difference between rates of DF in those under 10 years compared to those measured by Jarvis et al, suggesting that fluoride intake has remained high between 2009 and 2018. This suggests that residents may still be drinking from high-fluoride water sources or that there may be other aetiological factors playing a role in DF development.

One possible explanation is an inaccurate recall of primary drinking water sources. There may also be an element of social desirability or reporting bias in the answers given to the researchers, whereby the participants wish to give the ‘correct’ answer. Given that education programmes in Tindigani have promoted the use of piped water, there may be a desire by the respondents to be seen to be following this advice. As all of this information was self-reported it was not possible to accurately measure the length of exposure to high fluoride concentrations.

Alternatively, there might be significant utilisation of water sources other than the primary household water source – for example when out of the household or when the primary water source is unavailable. The household questionnaires revealed that if the piped water was inaccessible for some reason, for instance owing to a broken pipe or a road unpassable because of flooding, an alternative, typically high-fluoride, water source would be utilised.

Also, it may be that current drinking water practices have only been established recently and, therefore, have not yet made any significant positive impact on levels of DF in the population. As DF is a condition that depends, primarily, on fluoride consumption in the first few years of life and does not improve with time, patterns of DF in the population mainly reflect historical consumption of fluoride and longer-term studies would be required to assess effects of interventions.

Traditionally, ‘magadi’, a tenderising salt, often high in fluorine has been added to food in Tanzania and has previously been associated with DF and SF^21,32^. Jarvis et al. reported an OR of 6.05 (95% CI 2.07%, 17.67%, p<0.001) for magadi exposure in those with SF compared to those without but did not explore the link with DF.

This study did not seek to measure use of magadi or to ascertain other potential sources of dietary fluorine in Tindigani formally. However, from our informal discussions with residents, the majority reported using magadi regularly at some point in the past but not currently. However, this also may be subject to reporting bias. Further work would be required to investigate sources of dietary fluorine other than drinking water. Yoder et al. detected severe fluorosis in Kibosho in Northern Tanzania, an area with drinking water of low fluoride content, and cited high altitude, magadi and malnutrition as possible contributory factors^33^.

We observed a significant difference in the prevalence and severity of DF between children with deciduous and permanent dentition. These findings are in keeping with previous work, which has generally found fluorosis to be less prevalent and less severe in deciduous compared to permanent dentition^25,34^.

Development of deciduous dentition begins in utero and is usually completed by the age of four years as compared to permanent dentition which develops during the early years of life. Permanent dentition is, therefore, much more sensitive to elevated post-natal fluoride consumption than deciduous dentition^34-36^. In addition, it has been suggested that the placenta acts as a selective barrier for the passage of fluoride to the fetus, providing a measure of protection to the deciduous dentition from fluorosis caused by elevated antenatal maternal consumption of fluoride^37^. Fluoride has been detected in breast milk, albeit in low concentrations^38^.

We observed a significant difference in DF rates for younger children with deciduous dentition between those surveyed as part of the household survey and those in the school survey, with rates being higher amongst those in the school survey.

One possibility for this is that the household survey includes many children that do not attend school on a regular basis and, therefore, have lower exposure to the school's high-fluoride water source, which was measured to have a fluoride content of 12.6 mg/l. This is utilised for drinking water and cooking whilst the children are at school. Given the importance of fluoride exposure in the early years, securing a low-fluoride water source for the school should be a priority. However, further research would be required to ascertain the reason for the difference in DF rates between the two groups of children. In any event, in both cohorts, DF is almost universally present in permanent dentition.

DF remains endemic in Tindigani despite a reported shift away from use of high-fluoride groundwater and surface water sources to piped water for drinking. Although community water pipes were installed in the village, they initially only functioned for a short period. At the time of our research they were not in operation. This means that residents are faced with long journeys to access low-fluoride water sources from nearby villages or with reliance on nearby high-fluoride supplies. Since this study, the results have been communicated with Hai District Water Authority and arrangements were made with the aim of delivering clean low fluoride piped water to Tindigani. This has now been achieved.

There are several strengths to our study. This is the only follow-up study on DF in a high-fluoride area, therefore providing longitudinal data and evidence on the change in prevalence and severity over an extended period and the effects of providing de-fluoridated piped water on the development of DF. We studied a large sample size when compared to other DF prevalence studies in Sub-Saharan Africa^20^ thus increasing the accuracy and reliability of our data as well as its relevance in a wider context. The demographic data collected were supported by concurrent census data. We were therefore able to ensure our sample was representative of the village as a whole. It also means that our data could have relevance to other areas in the Hai district and across Tanzania that have similar population demographics and water fluoride concentrations.

However, there are also limitations. We were unable to survey the entirety of Tindigani, owing to heavy rainfall leaving some parts of the village inaccessible. Therefore, there is a possibility that the areas surveyed were not representative of the village as a whole, but we have no reason to suspect this.

The proportion of individuals for whom we were able to carry out dental examination varied with age and sex. For example, older males were more likely to be absent at the time of the survey. Since we know that there is a variation of DF prevalence with age, our overall prevalence figure must be treated with caution when comparing to prior research. Reports of the water sources utilised by households are potentially subject to recall and social desirability bias.

We did not investigate sources of dietary fluoride other than drinking water. There is an element of subjectivity in assessing DF. There may be variations between the grading performed by the assessors as part of this study, with that of prior research.

All water samples were taken during the rainy season. This allowed for direct comparison between the water samples however for a more accurate representation of average annual fluoride levels we would recommend a range of samples taken in both rainy and dry seasons. Previous research has shown a seasonal variation in fluoride water concentrations^30, 39–40^.

## CONCLUSION

This study supports existing data and provides evidence to drive interventions and policy change in affected areas. DF is a preventable public health problem and a high priority should, therefore, be placed on maintaining functioning pipes so that the residents of Tindigani can reliably access a low-fluoride water supply without making the time-consuming journey, by foot, to nearby villages. A particular priority is the provision of a lowfluoride supply to the village school so that exposure to excess fluoride is minimised during the early years of dental development and therefore reducing DF prevalence in future generations.
